# Graphic kinematics, visual virtual work and elastographics

**DOI:** 10.1098/rsos.170202

**Published:** 2017-05-24

**Authors:** Allan McRobie, Marina Konstantatou, Georgios Athanasopoulos, Laura Hannigan

**Affiliations:** 1 Cambridge University Engineering Department, Trumpington St, Cambridge CB2 1PZ, UK; 2 aktII, 100 St John Street, London EC1M 4EH, UK

**Keywords:** Maxwell reciprocal diagrams, Rankine reciprocal diagrams, Williot diagrams, graphic statics, kinematics, virtual work

## Abstract

In this paper, recent progress in graphic statics is combined with Williot displacement diagrams to create a graphical description of both statics and kinematics for two- and three-dimensional pin-jointed trusses. We begin with reciprocal form and force diagrams. The force diagram is dissected into its component cells which are then translated relative to each other. This defines a displacement diagram which is topologically equivalent to the form diagram (the structure). The various contributions to the overall Virtual Work appear as parallelograms (for two-dimensional trusses) or parallelopipeds (for three-dimensional trusses) that separate the force and the displacement pieces. Structural mechanisms can be identified by translating the force cells such that their shared faces slide across each other without separating. Elastic solutions can be obtained by choosing parallelograms or parallelopipeds of the appropriate aspect ratio. Finally, a new type of ‘elastographic’ diagram—termed a deformed Maxwell–Williot diagram (two-dimensional) or a deformed Rankine–Williot diagram (three-dimensional)—is presented which combines the deflected structure with the forces carried by its members.

## 1. Introduction

There has been much recent progress in the field of graphic statics, and in this paper we endeavour to extend that progress to encompass graphic kinematics. Until recently, graphic statics could be described as a simple geometrical method for finding the forces in a truss by drawing lines either perpendicular or parallel to the original bars. The geometry of any two-dimensional truss is given by its *form* diagram, and the corresponding *force* diagram is the collection of the force polygons at the structural nodes. The fact that there is a *closed* force polygon at each node corresponds to statical equilibrium. The form and force diagrams are reciprocal, in the sense defined by Maxwell in his pivotal paper of 1864 [1]. In a short paper, also in 1864, Rankine [2] described an analogous construction for three-dimensional trusses. There, the force in a bar of the structure is represented by the area of a polygon in the three-dimensional Rankine reciprocal diagram, and that polygon is orthogonal to the original bar. Nodal equilibrium then corresponds to the requirement that, for all bars that meet at a node, the reciprocal polygons combine to make a *closed* polyhedron.

Recent progress [3,4] has highlighted the link between these constructions and the Airy stress function for two-dimensional trusses, and the Maxwell–Rankine stress function for three-dimensional trusses. By a Maxwell–Rankine stress function, we mean one of the two three-dimensional stress functions defined in Maxwell’s classic 1870 paper [5], and specifically the one that corresponds to the Rankine reciprocal diagram.

For trusses, the stress function is necessarily continuous, but this has recently been generalized to discontinuous stress functions capable of describing the moments in frameworks with rigid connections. The theory for two-dimensional frames was presented in Williams & McRobie [6] and the generalization to three-dimensional frames was presented in McRobie [7]. This latter description is capable of describing all six stress resultants (an axial and two shear forces, and a torsional and two bending moments) in rather general three-dimensional frameworks with possibly curved members. Another innovation appeared in Zanni & Pennock [8] and McRobie [9] which showed how to combine form and force diagrams for a truss into a unified object, this being called the Maxwell–Minkowski diagram in two dimensions, and the Rankine–Minkowski diagram in three dimensions [9]. The resulting diagrams share much commonality with the graphical stress field (strut-and-tie) descriptions of forces within concrete structures, as described in Muttoni *et al.* [10], for example. For three-dimensional moment-carrying frames, the analogous unified object is the Corsican sum defined by McRobie [7].

This progress has, however, been largely restricted to the notion of statical *equilibrium*. While that is sufficient for lower bound plasticity approaches to truss design, there remains interest in elastic solutions, and this requires the appropriate treatment of compatibility (kinematics) and the inclusion of an elastic material law. Zanni & Pennock [8] showed how the unified form and force diagram also had a kinematic interpretation, placing emphasis on how the polygonal blocks of the diagrams could move relative to each other. While the present paper similarly considers how the blocks may move, the kinematic interpretation is extended to encompass the elastic deformations of the truss.

The starting point is the Williot diagram. This is the classical graphical construction created by Williot [11] for determining the displacements of a nodally loaded truss. The method is still often taught in university engineering courses where it is known simply as the displacement diagram. Williot’s method was extended by Mohr [12], leading to the Mohr–Williot diagram, which is easier to construct for more complex structures. The Williot and Mohr–Williot diagrams are described at length in Müller–Breslau’s two-volume textbook of the late ninteenth century [13,14] and more recently by Prakash Rao [15].

Throughout the paper, we shall combine two dual diagrams into a larger diagram, with each original node being replaced by a scaled version of its dual object. We shall refer to such a combination as a ‘Minkowski sum’ (denoted here by the symbol ⨁) even though it is only in simple cases that the final object is the projection of the Minkowski sum of two polyhedra.

We begin by showing how a displacement diagram can be created by translating pieces of the force diagram, giving visual expression to the principle of virtual work. Translations can be constructed which correspond to elastic displacements. Finally, the force diagram can be combined with the deflected shape, giving a figure containing all information. Throughout we consider two-dimensional trusses first, before showing the natural extension to three-dimensional trusses.

## 2. Maxwell–Williot diagrams

**Figure 1.**
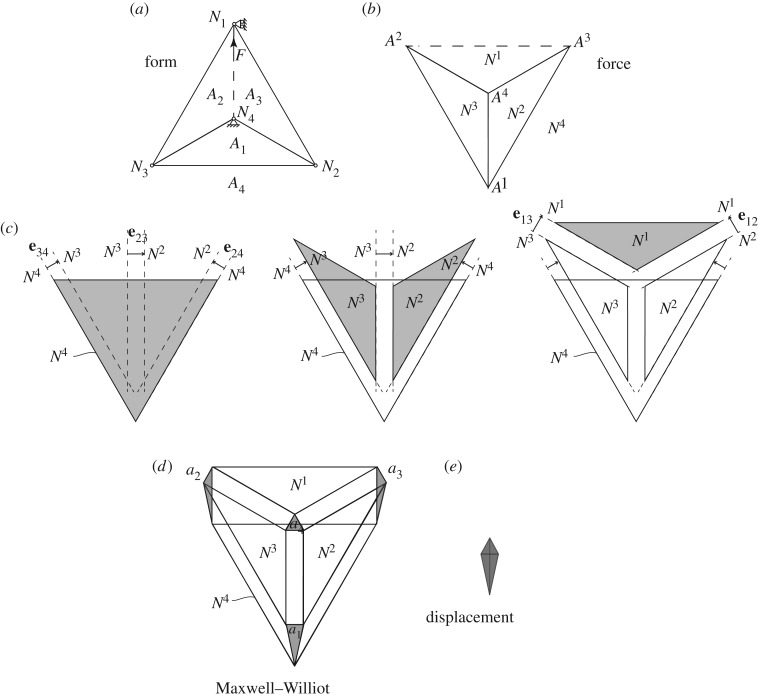
(*a*) The form diagram, showing the loaded structure. (*b*) The reciprocal force diagram. (*c*) The construction steps. Force triangles *N*^2^ and *N*^3^ are offset from force triangle *N*^4^ by distances equal to the extensions of the bars joining nodes *N*_2_,*N*_3_ and *N*_4_. Force triangle *N*^1^ is offset from *N*^2^ and *N*^3^. (*d*) The completed Maxwell–Williot diagram. (*e*) The displacement diagram so created.

Following Maxwell [1], we begin with perhaps the simplest example, illustrated in figure 1*a*, an equilateral triangular tension hoop with two compression radials. A force *F* is applied along the line of the missing third radial, as shown. For simplicity, the structure is symmetric and all angles are some multiple of 30°.

The loaded structure is the two-dimensional projection of a tetrahedron, and has the full K4 graph. The reciprocal diagram (figure 1*b*) is readily constructed, either purely graphically (drawing lines perpendicular to bars), or via the Airy stress function (plotting gradients of the tetrahedral stress function). Since tetrahedra are self-dual, the force diagram is also the projection of a tetrahedron.

The key to the construction is a reinterpretation of the Maxwell–Minkowski diagram. In its initial definition [8,9], a Maxwell–Minkowski diagram was simply a geometric combination of the form and force diagrams. Equivalently, we can view the construction as the act of translating one set of polygons apart in such a manner as to create the dual polygons within the nodal gaps. By extension, if we move the force polygons apart by arbitrary translations, we shall create an arbitrary set of polygons in the gaps. Joining the polygons so created will not, in general, lead to the form diagram. The diagram created will, however, have the same topology as the form diagram, and we shall take this created diagram to be the displacement diagram. That is, the nodes of the new diagram will define the nodal displacements of the structure relative to some origin, and a ‘bar’ of the new diagram will be the relative displacement between the two end nodes. The aim, generally, will be to move the force polygons apart in such a manner that the resulting displacement diagram, and thus the resulting bar extensions, satisfy some material law.

There are two possible conventions for two-dimensional reciprocal figures. In Maxwell’s convention, force vectors are drawn perpendicular to their corresponding bars, whereas in Cremona’s convention, forces and bars are drawn parallel. Here, we adopt Maxwell’s perpendicular convention. It follows that a bar extension **e**, being parallel to a bar, is thus drawn perpendicular to the reciprocal force vector.

For our example, the construction begins with the force triangle *N*^4^ which is reciprocal to the support node *N*_4_ which does not move. The radial bars connecting the central support *N*_4_ to the lower corners *N*_2_ and *N*_3_ are in compression. The triangles reciprocal to these nodes are thus moved inwards by distances **e**_24_ and **e**_34_ along the line of the original radials (and thus normal to the corresponding force vectors). These distances are the (compressive) extensions of the two radial bars.

That is, we move reciprocal triangles *N*^2^ and *N*^3^ by offsets **e**_24_ and **e**_34_ as shown in figure 1*c*. Since the nodes *N*_2_ and *N*_3_ are also connected by a tension bar which expands by **e**_23_, the reciprocal triangles *N*^2^ and *N*^3^ must be separated by this distance. Given the symmetry of the problem, the reciprocal triangles can thus be placed in their final locations.

The location of the final piece, reciprocal triangle *N*^1^, can now be determined similarly, via offsets **e**_12_ and **e**_13_ from triangles *N*^2^ and *N*^3^, as per figure 1*c*. The diagram is completed by connecting all the separated nodes by triangles. For example, in the pure force diagram (figure 1*b*), the point *A*^1^ at the base of the diagram is the meeting point of reciprocal triangles *N*^2^, *N*^3^ and *N*^4^. Once the reciprocal pieces are moved to their final positions, this single point *A*^1^ has been separated into a triangle, labelled *a*_1_ in figure 1*d*. There are four such triangles *a*_1_–*a*_4_, one at each of the formerly unseparated nodes *A*^1^–*A*^4^.

The completed diagram figure 1*d* we call a Maxwell–Williot diagram. It is the combination of the displacement diagram and the force diagram, in the same way that a Maxwell–Minkowski diagram [8,9] is a combination of the form and force diagrams. The displacement diagram, which is defined by the four triangles *a*_1_–*a*_4_, may now be extracted and combined into a single figure, as shown in figure 1*e*.

**Figure 2.**
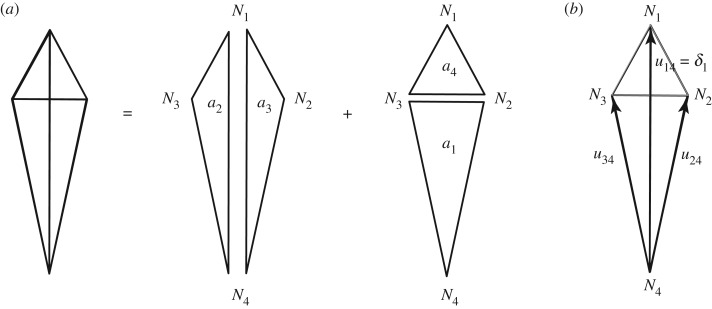
(*a*) The displacement diagram. (*b*) The vectors of nodal displacements **u** relative to the support node *N*_4_.

The displacement diagram is shown in more detail in figure 2*a*. The edges of this diagram give the relative displacements of the nodes. Since node *N*_4_ is a non-moving support, the displacements of the other nodes relative to *N*_4_ are their absolute displacements **u**.

A vector connecting nodes *i* and *j* gives the relative displacement **u**_*ij*_ of those nodes, and the component of that vector along the direction of the structural bar in the original form diagram is the extension **e**_*ij*_ of that bar.

## 3. Visual virtual work

**Figure 3.**
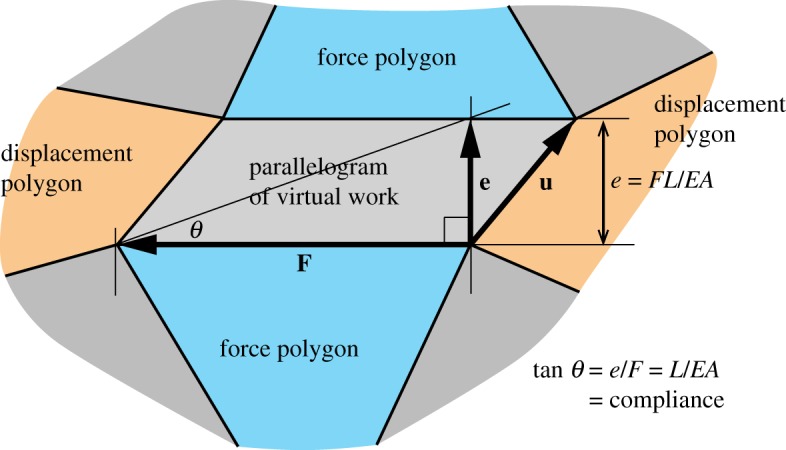
The parallelogram of virtual work that joins the force and displacement polygons.

The Maxwell–Williot diagram contains the polygonal pieces of the force and displacement diagrams stitched together by parallelograms. Figure 3 shows such a parallelogram. The area of the parallelogram is **F**×**u**, where the vector **F** is the force vector, drawn perpendicular to the original structural bar, and **u** is the relative displacement of the ends of the original bar. Trivially, **F**×**u**=**F**×**e**, where **e** is the bar extension.

The Maxwell convention has rotated the force through 90°. The cross product **F**×**e** thus equates to the dot product **F**_para_.**e**, where **F**_para_ is aligned parallel to the original bar. We thus obtain **F**×**e**=**F**_para_.**e**=*Fe*, where *e* is the component of relative displacement in line with the bar, which is thus the bar extension (with the usual assumptions of small displacement theory). That is, the area of the parallelogram equals *Fe*, which is the contribution of this bar to the overall virtual work.

If the bar, of length *L* and cross-sectional area *A*, is made of linear elastic material with Young’s modulus *E*, then *e*=*FL*/*EA* and the virtual work contribution is *Fe*=*F*^2^*L*/*EA*.

Figure 3 also illustrates that the (perpendicular) aspect ratio of the parallelogram is tan⁡θ=e/F=L/EA, the compliance of the bar.

In the simple example of the previous section, the Maxwell–Williot diagram consists of a double cover of a region of the plane by polygons. The total area of one cover must equal the total area of the other, and since each cover contains a cover of the force and displacement diagrams, it trivially follows that the area of parallelograms on one cover must equal that on the other. This is the visual expression of the virtual work equation:

3.1 $${\text{F}}_{\mathrm{ext}}.\delta_{\mathrm{ext}}=\sum_{\mathrm{bars}\;i}\int F_{i}\epsilon_{i}\;dL_{i}=\sum_{\mathrm{bars}\;i}\;F_{i}\frac{F_{i}L_{i}}{E_{i}A_{i}}.$$

In more general cases, the Maxwell–Williot diagram may be more than a double cover. The mental image would be of, say, a slightly inflated pita bread, made of rubber, that can be folded, creased or pleated and then flattened into any configuration. At general point, there will be an even number of layers. Given that the internal virtual work terms are *F*(*FL*/*EA*), these are always necessarily positive, due to the *F*^2^ factor. This means that all internal virtual work parallelograms must be contained in layers of the same orientation. For example, they must all lie in the odd-numbered layers of the folded pita, or they must all lie in the even-numbered layers, but there cannot be internal virtual work parallelograms in both odd- and even-numbered layers. This follows immediately from the construction and the equal areas argument.

There is an interesting special case where the structure is designed to carry an equal stress throughout. The width of the rectangles of the Maxwell–Minkowski combination of form and force diagrams then gives the amount of material required for each bar. If the stress is *σ*, and each bar has a width *b* and a through-the-paper depth of *t*, then the bar force *F*=*σbt*. In the Maxwell–Williot elastic displacement diagram, then perpendicular to the force *F* of the force diagram, the parallelograms have width *e*=*FL*/*EA*=*σbtL*/*Ebt*=*σL*/*E*. That is, the parallelograms have length *F* and width *σL*/*E*, this latter being a constant multiple of *L*. Notice though that Maxwell–Minkowski diagrams have rectangles of side length *F* by *L*, either of which can be rescaled arbitrarily. It may thus appear that a Maxwell–Minkowski diagram that uses a *σ*/*E* scaling of the force diagram will be a Maxwell–Williot diagram for the case of equal stress design. This is not so, however.

**Figure 4.**
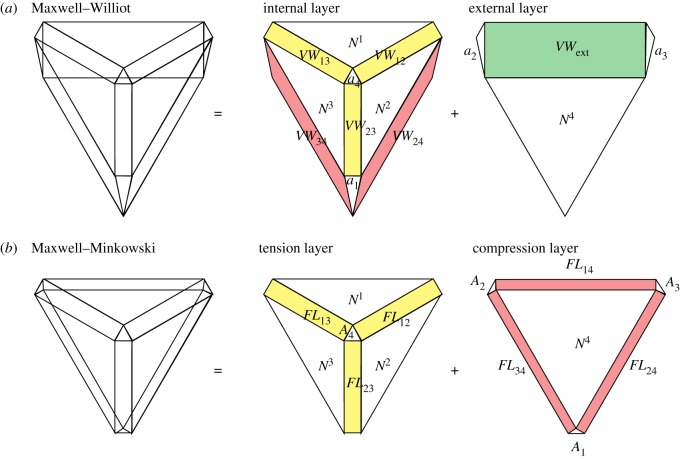
(*a*) The double cover of the Maxwell–Williot diagram. Since areas *N*^1^+*N*^2^+*N*^3^=*N*^4^ and *a*_1_+*a*_4_=*a*_2_+*a*_3_ (from the double covers of the individual form and force diagrams), it follows immediately that the external virtual work must equal the sum of the areas of parallelograms of internal virtual work. (*b*) The double cover of the Maxwell–Minkowski diagram.

This can be seen most clearly by comparing the Maxwell–Minkowski and Maxwell–Williot diagrams for our simple example, as shown in figure 4. In the Maxwell–Minkowski diagram, the areas of the rectangles correspond to the *F*.*L* terms of the Maxwell load path theorem. Those rectangles which correspond to tension members are thus in one layer, and those corresponding to compression members are in another layer. However, in the Maxwell–Williot diagram, all parallelograms corresponding to internal bars are in the same layer (figure 4*a*), irrespective of whether they carry tension or compression (due to the *F*^2^ factor in the virtual work equation). In this example, the rectangles *FL*_24_ and *FL*_34_ of the Maxwell–Minkowski corresponding to the two radial compression bars have, loosely speaking, been folded over in the Maxwell–Williot diagram to join the tension bars in the other layer, becoming parallelograms in the process.

It readily follows, from the load path theorem and the virtual work equation, that for a constant stress design in this simple case the displacement at the node *N*_1_ where the external load is applied must equal five times the extension of any one of the internal bars.

## 4. An alternative to the Mohr–Williot construction

It is well known (e.g. [15]) that the Williot construction can only be readily applied to simple structures such as cantilever trusses or structures with a high degree of symmetry, such as our earlier example. For more complicated structures, while a Williot diagram may exist, it cannot be constructed directly by the standard Williot procedure. This problem was solved by Mohr [12] via what is known as the Mohr–Williot construction. In essence, for a statically determinate structure, one ignores the support boundary conditions, choosing instead to keep one bar fixed in direction, and supported at one end. An artificial Williot diagram can then be constructed relative to these artificial boundary conditions, and this correctly gives the relative nodal displacements, but the absolute displacements do not meet the support conditions. To correct for this, Mohr showed how one could draw a scaled version of the structure adjacent to the artificial Williot diagram, and then find the absolute nodal displacements by reading between the two diagrams. The method is elegant, but it is far from intuitive. We thus describe an alternative that may be more suited to implementation into modern graphics processing software.

For the alternative, we simply create two artificial Maxwell–Williot diagrams which do not satisfy the boundary conditions, and then interpolate to obtain the one that does.

For the first, as per Mohr, we may pick boundary conditions where some bar is pinned at one end and can extend only along its length. For the second, we take the same bar, pinned at one end and free to not only extend but also to move some arbitrary amount sideways at the far end.

By linear interpolation of the sideways movement, the actual boundary conditions can be satisfied. We illustrate the procedure with an example of a truss bridge.

### 4.1. Example: the elastic deflections of a truss bridge

**Figure 5.**
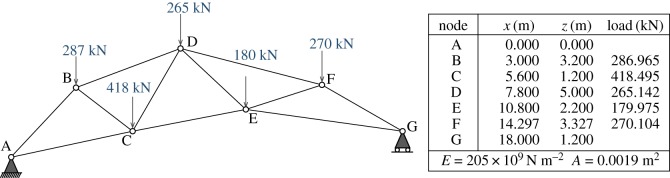
Bridge example.

**Figure 6.**
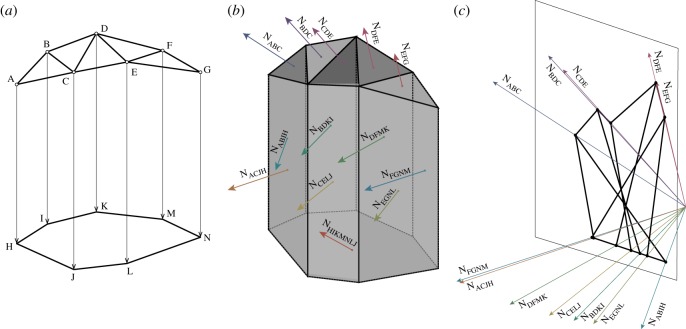
(*a*) A funicular polygon is added beneath the bridge to apply the loads. (*b*) The polyhedral Airy stress function, and the normals to its faces. (*c*) The force diagram defined by the normals.

**Figure 7.**
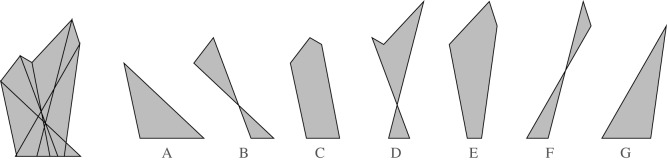
The polygonal pieces of the force diagram.

Figure 5 shows a nodally loaded asymmetric bridge. The standard graphic statics analysis of the equilibrium state is shown in figure 6, where a funicular polygon has been added below the structure, allowing the definition of a polyhedral Airy stress function whose normals define the force diagram. The polygonal pieces of the force diagram are shown in figure 7.

**Figure 8.**
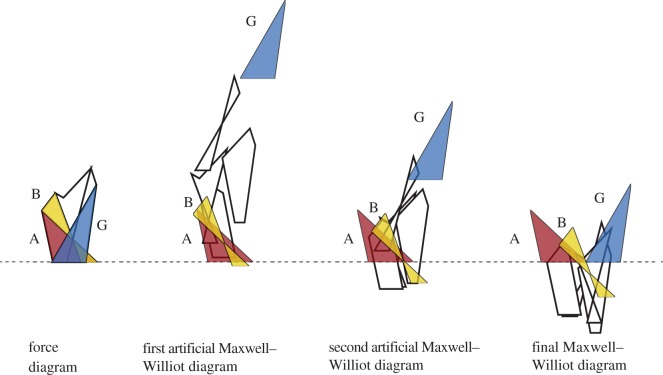
The force diagram and two artificial Maxwell–Williot diagrams for differing offsets of B parallel to edge AB. By linear interpolation, the offset of B that gives zero relative vertical displacement between supports A and G leads to the final Maxwell–Williot diagram.

The kinematic analysis begins by translating the polygonal pieces of the force diagram to offsets consistent with the bar extensions, as shown in figure 8. In the first instance, the bar AB is assumed to be fixed in direction, such that B can move only in the direction towards A. That is, dual polygon B moves perpendicular to the edge shared by polygons A and B. From this starting point, an artificial Maxwell–Williot diagram can be constructed for the whole structure. This gives the correct relative displacements of the nodes, but does not satisfy the global boundary conditions, since clearly the support G has moved vertically (as evidenced by the polygon dual to support node G being displaced vertically above the polygon dual to support node A). A second artificial Maxwell–Williot diagram is constructed assuming that B not only moves towards A, but moves perpendicular to that direction by some arbitrary amount. Again, the global boundary conditions are not satisfied because the polygon dual to G is still above that of A.

However, from the two artificial diagrams, it is a simple matter to linearly interpolate the lateral offset of B necessary for the polygons dual to A and G to remain at the same vertical height. That leads to the final Maxwell–Williot diagram that satisfies the global boundary conditions.

**Figure 9.**
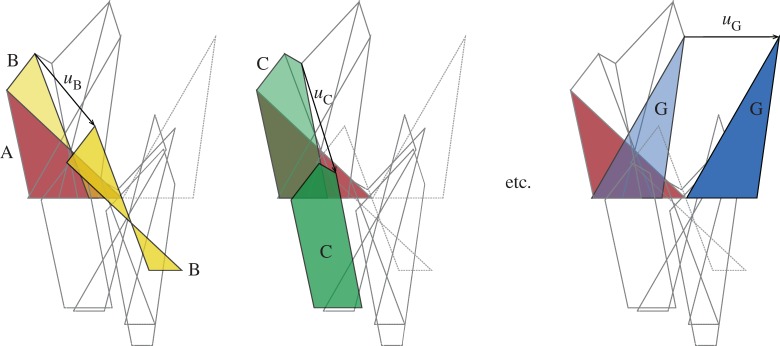
Nodal displacements are given by the translations of the pieces between the force diagram and the final Maxwell–Williot diagram.

**Figure 10.**
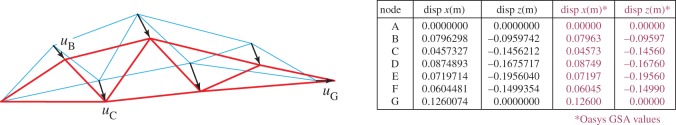
The deformed shape (red).

The nodal displacements are then given by the translations of the individual dual polygon pieces, as shown in figure 9. These are displayed in a more traditional fashion in figure 10, with the (exaggerated) deflected shape superimposed over the original structure.

It is to be emphasized here that the calculated nodal displacements, tabulated in figure 10, were obtained purely graphically, with no use of matrices. Also tabulated are the results of a standard matrix-based structural analysis software package, Oasys GSA [16], and it can be seen that the two are in agreement.

Given that structural finite-element packages—such as Oasys GSA—already exist for the solution of such problems, the question may arise as to what has been achieved by solving the problem graphically. The answer is that the graphical method provides a completely different methodology with which to approach structures. While it may appear to offer no particular comparative advantage in the analysis of a given structure, it is envisaged that advantages will accrue in a *design* rather than an *analysis* context. Analysis typically requires a structure to have been specified beforehand which can then be analysed, allowing members to be sized and deflections to be checked. Design, however, has a considerably broader remit. For example, at the initial concept phase, loads may be known but there may not yet be a structure to be analysed. It is at this stage where a graphical method which gives visual access to form, forces and displacements may prove to be of use. However, we leave such wider debate for the future.

## 5. Creating duals by moving pieces

The key to this paper is the recognition that cutting and translating the pieces of one diagram leads to a dual diagram. In two dimensions, we take any diagram which is the projection of a polyhedron and whose polygonal constituents thus give an at least double cover of a region of the plane. We then translate the polygonal pieces arbitrarily to new locations. An original node which was originally the corner point of several polygons thus becomes split into several nodes as the corners of the translated pieces separate. These now-separate corners thus define a polygon which is dual to the original node. The separated pieces and these new polygons form a general Maxwell–Minkowski diagram, with the original polygons and the new polygons connected by parallelograms. If the new polygons are extracted (or, equivalently, if we allow the original diagram to shrink to zero), then we obtain a diagram that is dual to the original diagram. If the original pieces are separated such that each edge is moved perpendicular to its original position, then the resulting dual is a Maxwell reciprocal diagram with dual edges perpendicular to original edges, and the connecting ‘parallelograms’ in the Maxwell–Minkowski diagram are actually rectangles. In the general case, though, dual edges need not be perpendicular. There are numerous cases where the non-perpendicular configuration is relevant. These include duals for no-shear grillage analysis [4], the discontinuous Airy stress function approach to moment-carrying frames [6] and the Maxwell–Williot combinations of force and displacement diagrams of this paper.

Moreover, the method of translating apart the pieces of one diagram to form its dual diagram readily generalizes to three-dimensional Rankine diagrams and their generalizations. In that case, the original diagram is the three-dimensional projection of a 4-polytope and the constituent polyhedra may be translated apart to create a dual polyhedron at each original node. This procedure has already been adopted in McRobie [7] where, for example, the loads on a gridshell roof were created by moving apart the polyhedral pieces of the original form diagram.

## 6. Mechanism analysis

To create the displacement diagram, pieces of the force diagram are translated apart, with each original edge becoming a parallelogram whose perpendicular width equals the bar extension. A parallelogram of zero width thus corresponds to a bar that does not extend. It follows that if it is possible to slide the force polygons along their shared edges (thereby creating parallelograms of zero width), then the dual diagram so created is the displacement diagram of a mechanism. We call the sliding of the polygonal pieces in such a manner a ‘sliding block mechanism’, although it is the pieces of the *force* diagram that ‘slide’, while the ‘mechanism’ is in the *form* diagram.

**Figure 11.**
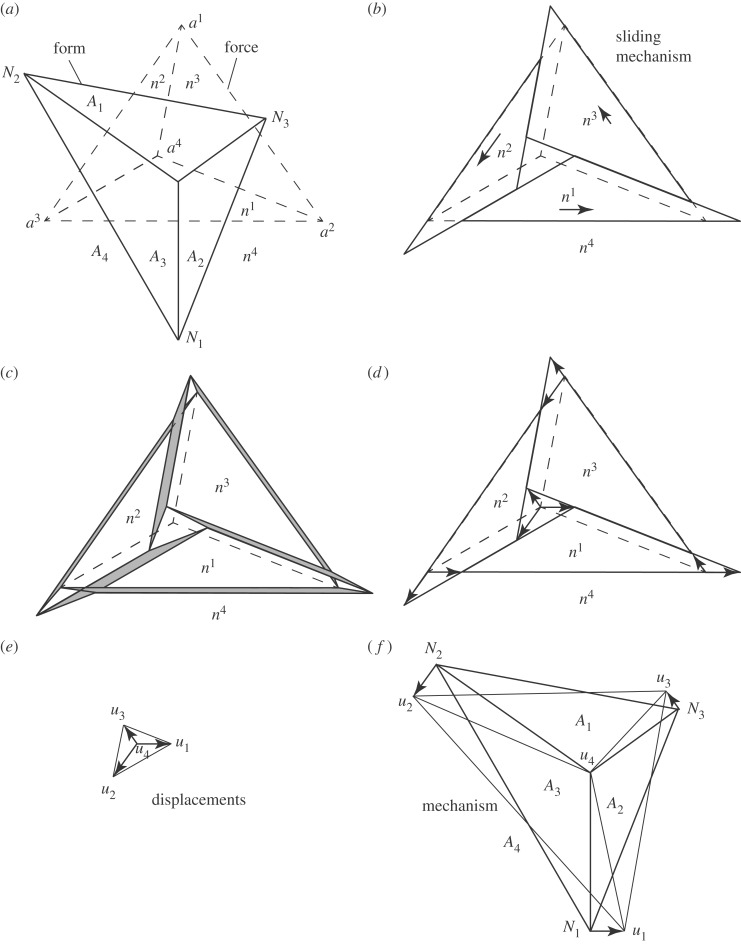
(*a*) Form and force diagrams. (*b*) Sliding mechanism of the force diagram. (*c*) Parallelograms artificially opened for clarity. (*d*) The displacements defined by the sliding. (*e*) The displacement diagram. (*f*) The structural mechanism.

Figure 11*a* shows the simplest example, based on the triangular truss of Fig. 1 of Maxwell 1864. Clearly a Maxwell–Williot diagram can be created by sliding the force polygons (without separating any edges) to the new locations shown in figure 11*b*. The parallelograms that conjoin the pieces have zero width, but have been shown artificially opened in figure 11*c* for visual clarity. The dual displacement diagram (figure 11*d*,*e*) can be extracted from each force vertex of the Maxwell–Williot diagram. Putting these displacement vectors at each node of the original form diagram shows the mechanism, in this case a simple rigid body rotation of the whole structure (figure 11*f*).

**Figure 12.**
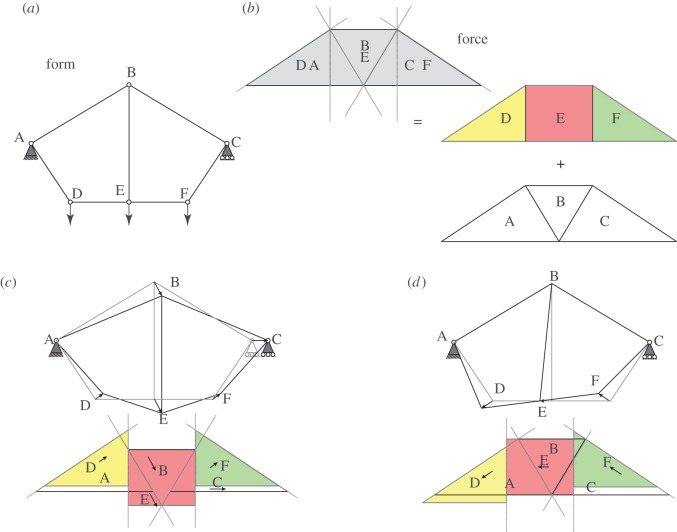
(*a*) Form diagram for the Beghini bridge variant. (*b*) The corresponding force diagram, decomposed to show the double cover. (*c*) The symmetric mechanism. (*d*) The antisymmetric mechanism.

Figure 12 shows a slightly less trivial example, based on the bridge geometry optimized by Beghini *et al.* [17]. As often occurs in graphic statics, the optimization process has a tendency to remove diagonal bracing bars, such that the optimal structure (for a given load case) contains numerous mechanisms which need to be identified.

The Maxwell–Williot perspective transforms the problem of finding inextensional mechanisms in a truss to one of finding sliding block mechanisms in the set of rigid polygonal blocks that constitute the force diagram. There is no contention that the new problem is any easier to solve than the original. However, the recognition that two seemingly separate problems are actually the same may prove to be of future use.

Figure 12*a*,*b* shows the form and force diagrams, while figure 12*c* shows the sliding construction for the symmetric mechanism. In the first instance, we consider the purely symmetric case, and then adjust for the boundary conditions later. The two triangular blocks corresponding to the supports A and C separate horizontally and symmetrically, allowing the central triangular wedge B to descend between them. The block E remains attached at the top edge of B and descends also. The two remaining blocks D and F must maintain face contact with their neighbours (D with A and E, and F with C and E) and thus move to the new locations shown. Finally, the fact that A is pinned, while C is on a roller support is addressed by giving the whole diagram a rightward displacement equal to the leftward displacement of A, such that A does not move. The translations of each block can then be extracted to obtain the nodal displacement diagram and these are superposed on the original form diagram to show the mechanism.

Figure 12*d* shows the construction for the antisymmetric mechanism. The abutment blocks A and C (and thus B remain unmoved. The central rectangle E can slide horizontally, maintaining contact with B along its upper edge. Again, the blocks D and F may then be located by means of the sliding contacts with their neighbours.

## 7. Maxwell–Williot deformed shapes

**Figure 13.**
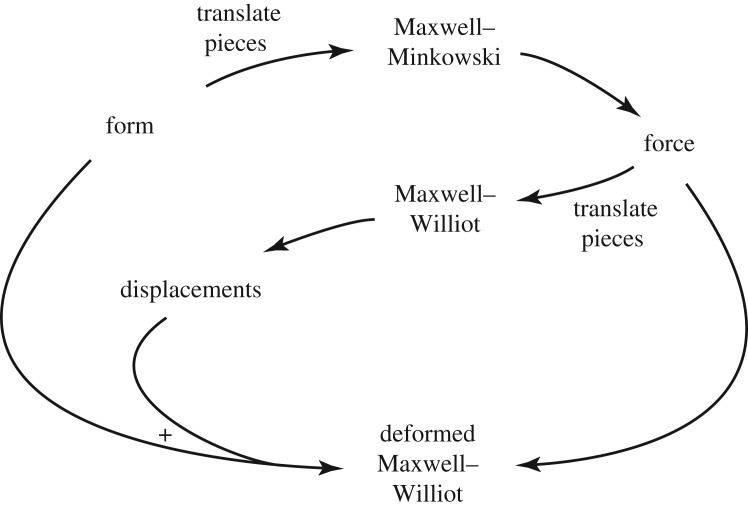
How the form, force and displacement diagrams combine to give the Maxwell–Minkowski, Maxwell–Williot and deformed Maxwell–Williot diagrams. (For three-dimensional trusses, ‘Maxwell’ is replaced by ‘Rankine’ throughout.)

The Maxwell–Minkowski diagram is a combination ⨁ of the structural form diagram and the Maxwell force diagram. The Maxwell–Williot diagram is likewise a sum ⨁ of the nodal displacement diagram and the Maxwell force diagram. Since the form diagram and displacement diagram necessarily have the same topology, it follows that we may take any linear combination *α***X**+*β***u**, where **X** are the nodal coordinates and **u** are the nodal displacements, and take the sum ⨁ of this combination with some scalar multiple *γ* of the Maxwell force diagram. Such a diagram we shall call a ‘deformed Maxwell–Williot diagram’. The relationship between the various diagrams is shown in figure 13.

**Figure 14.**
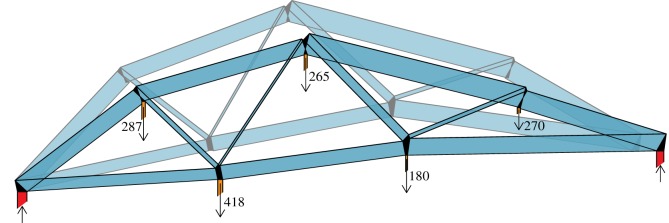
The deformed Maxwell–Williot diagram for the loaded asymmetrical truss bridge.

A general deformed Maxwell–Williot diagram, then, is the object (αX+βu)⨁(γF), where **F** are the coordinates of the force diagram relative to some arbitrary origin. If we choose *α*=*γ*=1, and *β* small, the resulting diagram will resemble the deflected structure, but with bars drawn thickened according to the force each carries. Such a diagram may be of use in providing a visual understanding of the structural behaviour. Figure 14 shows such a deformed Maxwell–Williot diagram for the earlier example of the loaded asymmetrical truss bridge.

This diagram contains all the information about the linear elastic response of the structure. It was created purely graphically, without use of matrices, matrix inversion or singular value decomposition. Since the phrase ‘graphic statics’ only covers the equilibrium part of this analysis, we adopt the phrase ‘elastographics’ to denote such diagrams that have been created graphically and yet satisfy equilibrium and compatibility with an elastic material law.

## 8. Rankine–Williot diagrams for three-dimensional trusses

The previous sections showed how to combine Maxwell and Williot diagrams for two-dimensional trusses. All of that analysis readily extends to three-dimensional trusses, using Rankine reciprocal diagrams to obtain Rankine–Williot diagrams.

**Figure 15.**
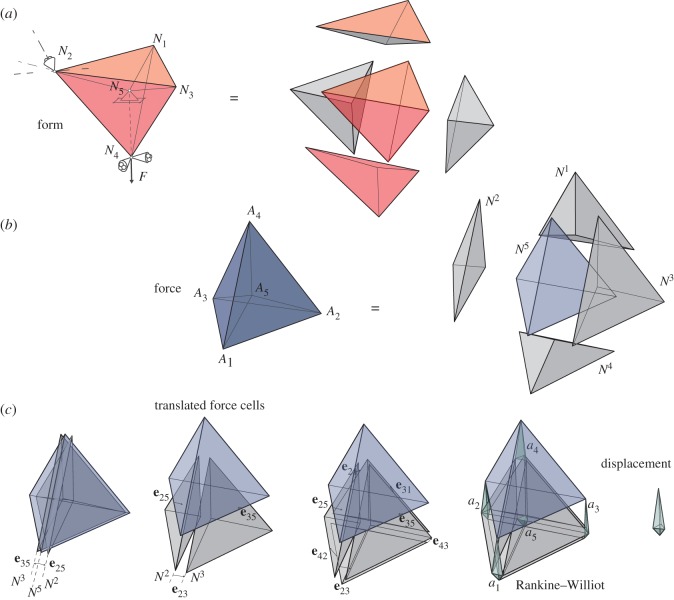
(*a*) A regular tetrahedron with radial spokes, loaded along the line of the missing spoke *N*_4_–*N*_5_. (*b*) The force diagram and its component tetrahedra. (*c*) Translations of the pieces of the force diagram in accordance with the bar deformations, and the resulting Rankine–Williot diagram from which the nodal displacement diagram may be extracted.

Figure 15 shows the simplest three-dimensional example, a regular tetrahedron connected by radial bars to a central support. One of the radial bars is replaced by an external force acting along the line of the missing bar. The bars and support conditions have been chosen to give a symmetrical solution. The problem is essentially the three-dimensional version of the example of §2.

**Figure 16.**
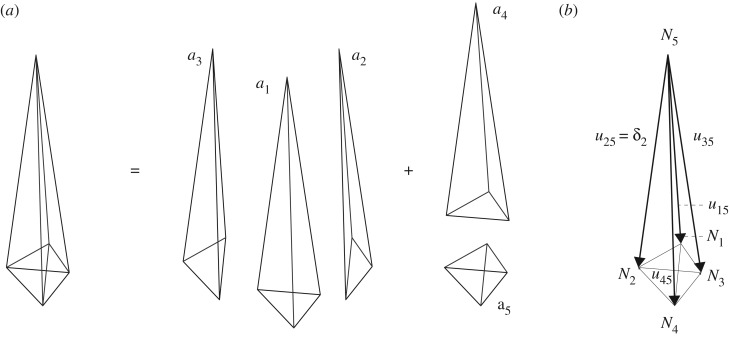
(*a*) The relative displacement diagram of the loaded simplex. (*b*) The absolute nodal displacements (relative to node *N*_5_).

Figure 15*a*,*b* shows the form and force diagrams, and their constituent polyhedra. Figure 15*c* shows how the tetrahedral pieces of the force diagram are translated to offsets relative to *N*^5^, the force tetrahedron that is dual to the central support node of the structure. Figure 15*d* shows the final Rankine–Williot diagram when all force tetrahedra have been appropriately translated, and from this the nodal displacements can be extracted. These are shown magnified in figure 16.

**Figure 17.**
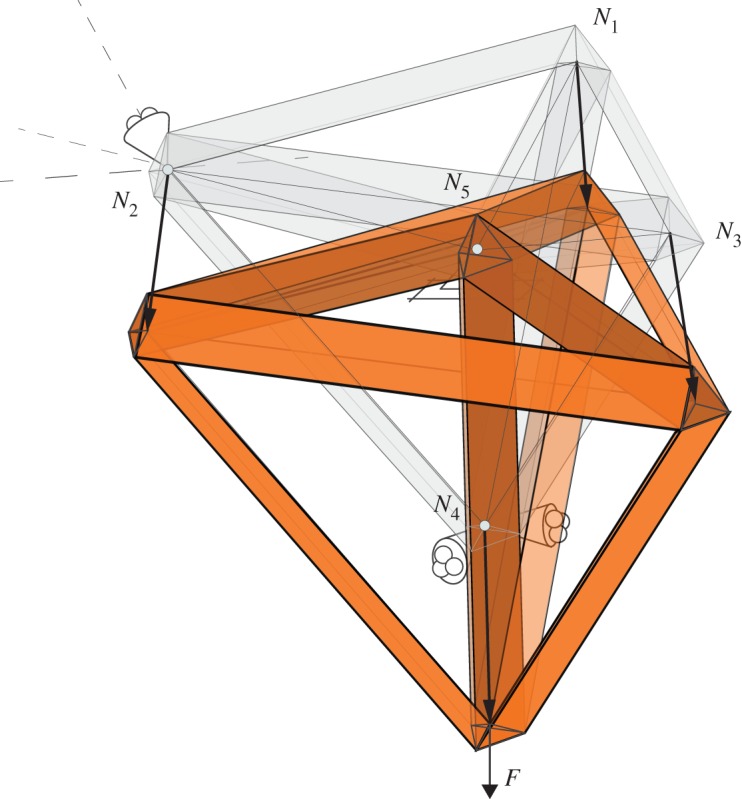
The deformed Rankine–Williot diagram showing the deflected shape of the structure.

Finally, we may take a suitable linear combination of the original form diagram with the calculated displacements and then take the Minkowski sum with the force diagram to obtain the deformed Rankine–Williot diagram (see figure 17). This is a three-dimensional elastographic diagram that contains all the information about the elastic structural behaviour of the loaded truss.

## 9. Three-dimensional truss mechanisms

McRobie *et al.* [4] described how there is an isomorphism between states of self-stress in one two-dimensional truss diagram and the mechanisms in the two-dimensional reciprocal diagram, a result previously noted by Crapo & Whiteley [18] (H Crapo, W Whiteley 1994, unpublished draft). This was demonstrated by incrementing the Airy stress function over one diagram, thereby moving reciprocal bars to a small offset while remaining parallel to their original directions. These incremental offsets then defined a displacement diagram which, when rotated by 90°, corresponds to a mechanism in the original truss. While this is valid in two dimensions, it does not generalize to three dimensions, because there is no unique axis about which to apply the 90° rotation. However, the earlier identification of inextensional structural mechanisms with sliding block mechanisms of the force diagram for two-dimensional trusses readily generalizes to three dimensions: instead of sliding polygonal pieces of the two-dimensional Maxwell reciprocal, one can slide polyhedral pieces of the three-dimensional Rankine reciprocal.

**Figure 18.**
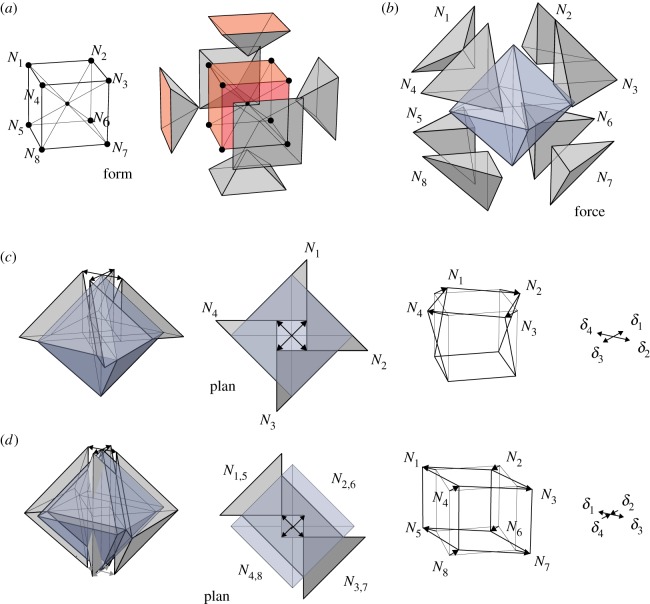
(*a*) The form diagram of a spoked cube. (*b*) The force diagram is a spoked octahedron. (*c*) A mechanism of the spoked cube. (*d*) A mechanism of the unspoked cube.

This is illustrated in figure 18*a*, which shows a ‘spoked cube’ truss. Although this structure can maintain a state of self-stress with, say, the spokes in tension and the cube members in compression, there are numerous infinitesimal mechanisms in addition to the rigid body rotations.

The Rankine reciprocal is a spoked octahedron (figure 18*b*) consisting of eight tetrahedra nestled inside the surrounding octahedron. Figure 18*c* shows a sliding block mechanism involving four tetrahedra, and the corresponding displacements of the three-dimensional truss mechanism. Although the tetrahedra have been translated, all faces maintain contact.

In contrast, figure 18*d* shows a sliding block mechanism where the inner tetrahedra do not maintain face contact with the outer octahedron. This implies that there is extension of the radial spokes, and so this cannot correspond to a truss mechanism of the spoked cube. However, if we choose the spokes to have infinite flexibility, then we may ignore the spoke extensions, and the remainder is a sliding block mechanism that corresponds to a three-dimensional truss mechanism of the unspoked cube.

The idea that spokes can be included, and then assumed to have infinite flexibility (and thus ignored), means that the kinematics of many objects can now be addressed which may previously have seemed inaccessible to graphic analysis. The idea is closely linked to the Zero Bar concept for structural equilibrium introduced in McRobie [19], where bars could have polygonal cross sections of zero oriented area, and thus carried no force. In both cases, the object is coned, but the radials to the pole do not contribute to the physics.

**Figure 19.**
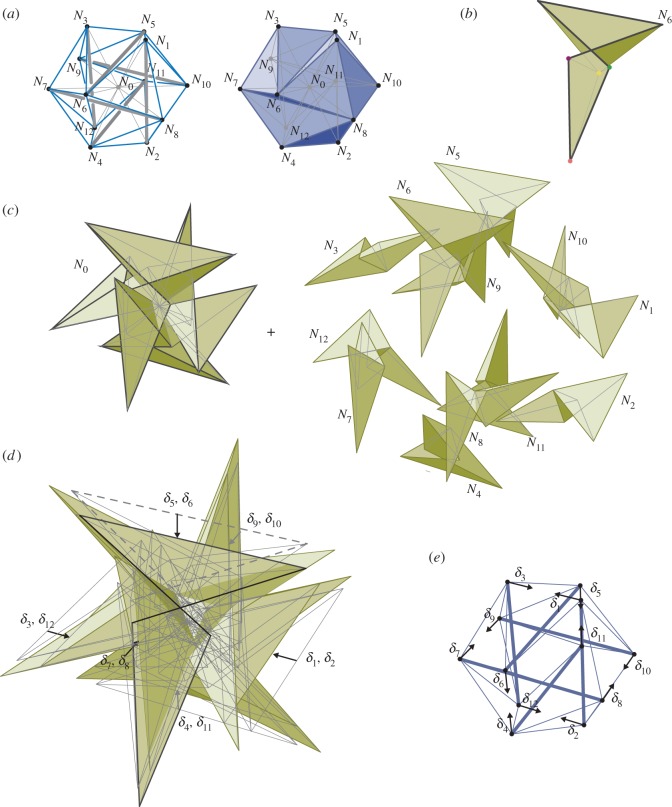
(*a*) The Jessen icosahedral tensegrity. (*b*) One of the cells of the Rankine reciprocal. (*c*) All cells of the Rankine reciprocal. (*d*) Sliding block mechanism of the reciprocal cells, but allowing faces normal to radial spokes to lose contact. (*e*) The three-dimensional truss mechanism.

Figure 19*a* shows a Jessen icosahedral tensegrity. The structure has been coned via ‘Zero Bars’ to allow a Rankine reciprocal to be constructed (figure 19*b*,*c*). That is, each vertex is connected by a spoke to a central node, but the spokes have zero area and thus carry no force. The structure possesses a state of self-stress that is restricted to the outer icosahedron, with the spokes remaining unstressed. The construction is described in more detail in McRobie [9,19]. There is no sliding block mechanism for the full Rankine reciprocal of the spoked icosahedron. However, if the faces normal to the spokes are allowed to lose contact with the enclosing (and rather elaborate) icosahedron, then a sliding block mechanism exists, as shown in figure 19*d*. That is, if spokes are assumed to have infinite flexibility, then the sliding block mechanism in figure 19*d* corresponds to an infinitesimal three-dimensional truss mechanism of the unspoked tensegrity. The translation of the force cells, when drawn at their dual nodes, illustrate the infinitesimal structural mechanism in traditional form (figure 19*e*).

## 10. Summary and conclusion

This paper has described a new concept which we call ‘elastographics’. This is achieved by combining Williot displacement diagrams (which describe structural kinematics) with two-dimensional Maxwell or three-dimensional Rankine reciprocal diagrams (which describe equilibrium), thereby providing purely graphical solutions of the elastic structural behaviour of nodally loaded two- and three-dimensional trusses. In this scheme, the various contributions to the virtual work equation are made visible, and this may prove to be advantageous in future structural optimization schemes. An identification has been made between infinitesimal structural mechanisms of the truss and ‘sliding block mechanisms’ of the reciprocal force diagram. Finally, deformed Maxwell–Williot and deformed Rankine–Williot diagrams have been described which give full visual expression to the elastic solutions.

The overall intention is that these new descriptions and perspectives will allow the substantial recent progress in graphic statics to be extended into wider spheres of structural engineering. The use of graphical software is now ubiquitous among architects and building designers for the visualization and manipulation of structural form. The extensions described here allow solution, visualization and manipulation of the structural performance using the same graphical tools, and provide a natural alternative paradigm for structural design.
